# Coagulation biomarkers and prediction of venous thromboembolism and survival in small cell lung cancer: A sub-study of RASTEN - A randomized trial with low molecular weight heparin

**DOI:** 10.1371/journal.pone.0207387

**Published:** 2018-11-09

**Authors:** E. Gezelius, A. Flou Kristensen, P. O. Bendahl, Y. Hisada, S. Risom Kristensen, L. Ek, B. Bergman, M. Wallberg, U. Falkmer, N. Mackman, S. Pedersen, M. Belting

**Affiliations:** 1 Department of Clinical Sciences, Division of Oncology and Pathology, Lund University, Lund, Sweden; 2 Department of Hematology, Radiophysics and Oncology, Skane University Hospital, Lund, Sweden; 3 Department of Clinical Biochemistry, Aalborg University Hospital, Aalborg, Denmark; 4 Department of Clinical Medicine, Aalborg University, Aalborg, Denmark; 5 Department of Medicine, Division of Hematology and Oncology, Thrombosis and Hemostasis Program, University of North Carolina at Chapel Hill, Chapel Hill, NC, United Sttaes of America; 6 Department of Respiratory Medicine, Lund University Hospital, Lund, Sweden; 7 Department of Lung Medicine, Sahlgrenska University Hospital, Gothenburg, Sweden; 8 Department of Oncology, Aalborg University Hospital, Aalborg, Denmark; University of Patras, GREECE

## Abstract

Coagulation activation and venous thromboembolism (VTE) are hallmarks of cancer; however, there is an unmet need of improved biomarkers for individualized anticoagulant treatment. The present sub-study of the RASTEN trial was designed to explore the role of coagulation biomarkers in predicting VTE risk and outcome in a homogenous cancer patient population. RASTEN is a multicenter, randomized phase-3 trial investigating the survival effect of low molecular weight heparin enoxaparin when added to standard treatment in newly diagnosed small cell lung cancer (SCLC) patients. Plasma collected at baseline, during treatment, and at follow-up was used in this *ad hoc* sub-study (*N* = 242). Systemic coagulation was assessed using four assays reflecting various facets of the coagulation system: Total tissue factor (TF); extracellular vesicle associated TF (EV-TF); procoagulant phospholipids (PPL); and thrombin generation (TG). We found small variations of biomarker levels between baseline, during treatment and at follow-up, and appeared independent on low molecular weight heparin treatment. Overall, none of the measured biomarkers at any time-point did significantly associate with VTE incidence, although increased total TF at baseline showed significant association in control patients not receiving low molecular weight heparin (*P* = 0.03). Increased TG-Peak was significantly associated with decreased overall survival (OS; *P* = 0.03), especially in patients with extensive disease. Low baseline EV-TF predicted a worse survival in the low molecular weight heparin as compared with the control group (HR 1.42; 95% CI 1.04–1.95; *P* = 0.03; *P* for interaction = 0.12). We conclude that the value of the analyzed coagulation biomarkers for the prediction of VTE risk was very limited in SCLC patients. The associations between TG-Peak and EV-TF with patient survival and response to low molecular weight heparin therapy, respectively, warrant further studies on the role of coagulation activation in SCLC aggressiveness.

## Introduction

Oncogene activation and the tumor microenvironment induce a hypercoagulable state and an increased risk of venous thromboembolism (VTE) [1–3], which are hallmarks in malignancy and major contributors to cancer-associated mortality and morbidity [4–6]. It is important to find predictive biomarkers to identify patients who may benefit from prophylactic anticoagulant treatment, not only in terms of preventing thrombotic events but also to improve survival.

Thrombin is a key factor in coagulation activation [7], and its generation has been widely studied to unravel the mechanisms of cancer-associated VTE. Thrombin generation (TG), as determined by the calibrated automated thrombogram (CAT) assay, has recently gained interest as a biomarker of disease aggressiveness as well as VTE risk in cancer [8,9]. Cancer-associated hypercoagulability may in part be explained by the activation of tissue factor (TF), which is the main initiator of coagulation [6,10,11]. Tumor TF expression has been correlated to increased risk of VTE and metastatic disease, indicating that TF has direct clinical implications both in tumor progression and VTE development [12]. TF can be released from tumor cells in the form of extracellular vesicles (EV-TF) or as an alternatively-spliced from lacking the transmembrane domain [13,14]. Previous studies in small patient cohorts have found some evidence for a correlation of EV-TF activity with VTE in pancreatic cancer but not in other forms of cancer, including gastric, brain, lung and ovarian cancers [15–18]. Procoagulant phospholipids (PPL) represent another important factor for coagulation activity with potential to predict VTE risk. PPL are exposed on the outer membrane leaflet of EVs and provide a negatively charged surface for the decryption of TF, assembly of coagulation complexes, and thrombin formation [19,20].

Apart from its antithrombotic properties, pre-clinical evidence indicates that heparin and low molecular weight heparin (LMWH) have direct tumor-inhibiting effects via *e*.*g*. inhibition of angiogenesis and metastasis [21–23]. The potential antitumoral effects have been further investigated in the clinical setting. A few early studies showed survival benefits when anticoagulants were administered prophylactically to cancer patients, particularly in small cell lung cancer (SCLC) [24–25]. However, more recent randomized trials have not been able to show survival advantage with LMWH in lung cancer [26,27]. This includes a phase III trial in which a homogenous population of SCLC patients were randomized to receive standard treatment with or without the addition of LMWH (RASTEN) [27].

In the present sub-study of the RASTEN trial, our aim was to directly compare the potential utility of coagulation-related biomarkers for the prediction of VTE risk in SCLC patients using a comprehensive approach that includes total TF, EV-TF, TG, and PPL. Also, we addressed correlations between coagulation biomarkers and patient survival to elucidate the potential role of coagulation activation in SCLC aggressiveness.

## Materials and methods

### RASTEN clinical trial

A full description of the study design has been reported previously [27]. In brief, RASTEN (ClinicalTrials.gov: NCT00717938) is an international, prospective, open-label trial in patients with newly diagnosed SCLC of all stages, WHO performance status 0 to 3 and standard coagulation parameters within normal ranges. Patients were randomized 1:1 between a control arm receiving standard treatment and an intervention arm receiving standard treatment with the addition of LMWH enoxaparin given at 1 mg/kg as daily subcutaneous injections, starting on day 1 of chemotherapy and continued throughout the duration of the chemotherapy regimen. Standard therapy included a platinum compound and a topoisomerase inhibitor administered for 4–6 cycles according to local guidelines. Radiotherapy was given depending on disease extent and response to chemotherapy, following local protocols. Written, informed consent was obtained from all study participants. The study was carried out according to the ICH/GCP guidelines, in agreement with the Helsinki declaration and with approval from the regional ethics committee at Lund University, Sweden.

### Patient selection and plasma sampling

Plasma was collected at baseline, prior to the third chemotherapy cycle and at a 2 months’ follow-up visit according to the study protocol. Blood samples were collected in sodium citrate and EDTA tubes, centrifuged at 2000 x *g* for 15 min at room temperature (RT) and stored in a -80°C freezer. The present biomarker cohort was established at the cut-off date of November 1^st^ 2013, consisting of the first consecutive 292 patients.

### Total tissue factor

Total TF was determined in patient EDTA-plasma at baseline using the Proseek Multiplex CVD^96x96^ panel (Olink Bioscience, Uppsala, Sweden), as previously described [28]. The assay is based on proximity extension assay (PEA) technology, which provides high sensitivity and specificity based on the binding of oligonucleotide-labeled antibody probe pairs to their specific target protein, generating a PCR-amplified DNA template, which is proportional to the initial antigen concentration as quantified by real-time qPCR. Four internal and three negative controls were used to calculate the lower limit of detection (LOD) for each protein.

### Tissue factor activity associated with extracellular vesicles

EV-TF activity was determined as described previously [29]. Briefly, EVs were pelleted by centrifugation at 20 000 x *g* for 15 min and mixed with human anti-TF-antibody (HTF-1; BD Pharmingen, CA, USA) or mouse control IgG antibody (BD Pharmingen, CA, USA) at RT for 15 min, followed by addition of coagulation factors VIIa (10 nM) and X (300 nM) as well as CaCl_2_ (10 mM) in a 96-well plate. A standard curve of recombinant human TF Innovin (0–55 pg/ml, Siemens, Germany) was applied to the plate, which was then incubated for 2 h at 37°C. FXa generation was terminated by EDTA (25 nM), after which Pefachrome FXa (Pentapharm, Switzerland) substrate was added for 15 min at RT, and absorbance was measured at 405 nm using a VERSAmax microplate reader (Molecular Devices Corp., CA, USA). TF-dependent FXa generation was determined by subtracting the obtained values in hTF-1 wells from values in control IgG wells.

### Procoagulant phospholipid assay

PPL activity was measured per manufacturer’s instructions using Procoag-PPL (STAGO inc., France), which is a FXa-based clotting method that utilizes phospholipid-depleted plasma to assess the activity of PPL in samples. Briefly, 25 μL sample plasma was added to a cuvette containing 25 μL human phospholipid-depleted plasma, and incubated for 2 min at 37°C. Subsequently, pre-heated XACT-reagent (STAGO inc., France) containing FXa and Ca^2+^ was added, and clotting time was determined based on the motion of a spherical steal ball. Notably, a short clotting time indicates an increased PPL activity.

### Thrombin generation assay

The TG capacity was determined using a modified version of the calibrated automated thrombogram assay [30]. Since LMWH interferes with the assay, analyses at cycle 3 included patients only in the control arm. Briefly, 65 μL sodium citrate plasma was mixed with 20 μL of either trigger (1 pM TF and 4 μM phospholipids; PPPlow, Thrombinoscope BV, Netherlands) or calibrator solution. Samples were heated to 37°C and TG was initiated using 20 μl pre-heated FluCa buffer containing a fluorescence substrate and CaCl_2_ (Thrombinoscope BV, Netherlands). Fluorescence intensity was read over 45 min with a 390/460 excitation/emission filter set and TG curves were generated using Thrombinoscope software version 5.0 (Thrombinoscope BV, Netherlands). Three established TG parameters were validated through statistical analyses; endogenous thrombin potential (TG-ETP), peak height (TG-Peak), and time to peak (TG-ttPeak).

### Statistical analyses

The statistics programs SPSS v22, Stata 15.1, and R 3.3.0 were used for data analysis and graphics. Correlation between pairs of biomarkers was quantified using Spearman’s rank correlation coefficient and visualized in a heat map using the R-package ggplot2. Non-parametric tests were used for comparisons of biomarker levels in different subgroups (Mann-Whitney test) and over time (Wilcoxon matched-pairs signed rank test). The Kaplan-Meier method was used to estimate survival probabilities and the evidence for difference in survival between groups formed by categorization of a biomarker was evaluated using a logrank test or, for three ordered categories, a logrank test for trend. Cox regression was used to quantify the corresponding biomarker effects on survival as hazard ratios. A multivariable Cox model with an interaction term was used to test for differential treatment effect in subgroups based on a biomarker. Time zero was defined as date of diagnosis in survival analyses including biomarkers measured only at baseline. For analyses at later time points, time zero was shifted to the corresponding sampling date (landmark analysis). The *P*-values in this exploratory study have not been adjusted for multiple testing. The reader should keep this in mind when interpreting the level of evidence for each test performed. The *P*-values should not be compared to the often used cut-off 5%, but rather be seen as continuous measures of evidence against the null hypotheses.

## Results

### Study population

**Fig 1:**
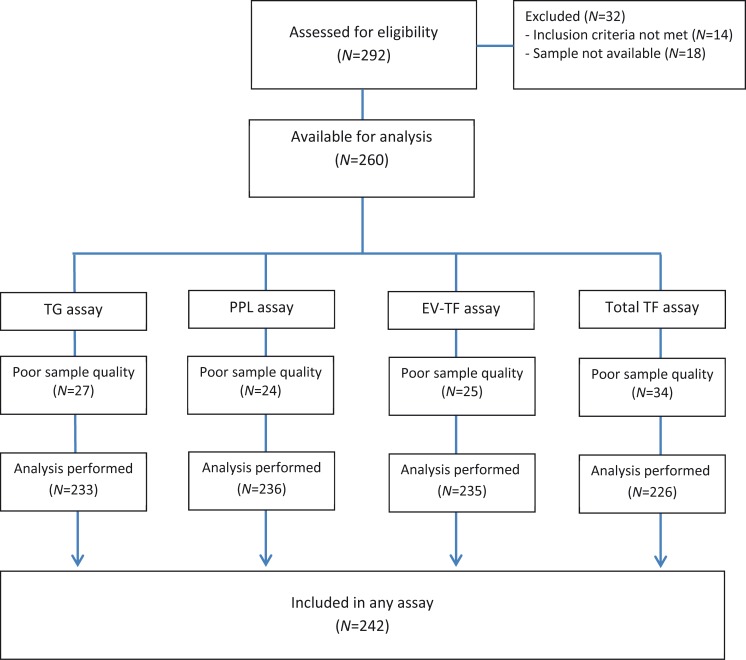
Consort diagram of the study population.

By November 2013, blood samples had been collected for the first 292 patients enrolled in the RASTEN trial (Fig 1). Eight patients were excluded as further investigations showed other histology than SCLC, and six patients did not fulfil the inclusion criteria. Samples were unavailable in 18 cases. For each of the assays, a varying number of samples were excluded due to insufficient volumes or hemolysis. In total, blood samples from 242 patients were included in the present coagulation biomarker cohort, 115 in the LMWH and 127 in the control arm. Baseline characteristics were comparable between both study arms (Table 1). Median follow-up was 276 days for patients still alive. Median overall survival (OS) was 9.9 and 10.9 months (*P* = 0.08), with 1-year survival rates of 41% and 45% in the LMWH and control arms, respectively. Fifteen patients developed VTE, of which twelve and three patients were in the control and LMWH arm, respectively.

**Table 1:** Baseline characteristics of the study population.

	LMWH *N* = 115	Control *N* = 127
**Age, years**		
Mean ± SD	66 ± 8.1	67 ± 8.7
**Gender, *N* (%)**		
Female	64 (56)	72 (57)
Male	51 (44)	55 (43)
**Performance status, *N* (%)**		
0–1	81 (70)	91 (72)
2–3	34 (30)	36 (28)
**Disease stage, *N* (%)**		
Limited	47 (41)	52 (41)
Extensive	68 (59)	75 (59)
**Biochemistry, median (IQR)**		
Hemoglobin, g/L	134 (122–144)	133 (122–141)
Leukocyte count, x10^9^/L	9.4 (7.3–12.2)	9.6 (7.0–12.8)
Platelet count, x10^9^/L	336 (263–445)	309 (257–419)
Sodium, mmol/L	138 (135–140)	138 (134–141)
Potassium, mmol/L	4.1 (3.9–4.4)	4.3 (4.0–4.5)
Serum creatinine, μmol/L	65 (56–73)	66 (55–79)
aPTT, s	32 (28–36)	32 (28–35)
**Overall survival**		
Median, months	9.9	10.9
12 months, % (95% CI)	41 (32–50)	45 (36–53)
**VTE events, *N* (%)**	3 (3)	12 (9)

SD = Standard deviation; IQR = Interquartile range; aPTT = Activated partial thromboplastin time; VTE = Venous thromboembolism.

### Coagulation biomarkers depending on disease extent and LMWH treatment

As expected in this randomized trial, all coagulation biomarkers were comparable in the control and LMWH treatment arms at baseline (Table 2**)**. TG parameters, PPL activity, and EV-TF showed small variations between baseline, during treatment and at follow-up, and appeared independent on LMWH treatment (Table 2). Baseline EV-TF, but none of the other biomarkers, was significantly higher in patients with ED as compared with LD (*P* = 0.04) (S1 Table). The distribution of EV-TF levels was particularly skewed with a large proportion of samples below or near the detection limit. Hence, the patient group was dichotomized according to the 75th percentile, representative of an EV-TF cut-off value of 0.32 pg/ml, which revealed that 74% in the upper quartile had ED.

**Table 2:** Biomarkers at baseline, prior to cycle 3 and at 2 months’ follow up, for all patients and by treatment arm.

	All patients		LMWH arm		Control arm		
	Median (IQR)	*N*	Median (IQR)	*N*	Median (IQR)	*N*	*P*-value^a^ (LMWH *vs*. Control arm)
**EV-TF (pg/ml)**							
Baseline	0.19 (0.08–0.32)	235	0.21 (0.08–0.32)	110	0.18 (0.06–0.32)	125	0.43
Cycle 3	0.19 (0.09–0.31)	193	0.20 (0.10–0.30)	94	0.19 (0.08–0.32)	99	0.99
Follow up	0.15 (0.07–0.26)	130	0.13 (0.08–0.25)	65	0.16 (0.05–0.28)	65	0.75
**TG-Peak (nM)**							
Baseline	219 (175–263)	233	227 (171–268)	111	218 (176–262)	122	0.73
Cycle 3^b^	-		-		264 (203–351)**	98	-
Follow up	259 (187–337)**	115	277 (195–340)*	61	252 (168–332)*	54	0.51
**TG-ttPeak (min)**							
Baseline	9.8 (8.5–11.3)	233	9.8 (8.7–11.2)	111	9.9 (8.3–11.6)	122	0.93
Cycle 3^b^	-		-		9.2 (8.0–10.5)*	98	-
Follow up	9.7 (8.3–11.2)	115	9.7 (8.0–10.9)	61	9.6 (8.5–12.3)	54	0.59
TG-ETP (nM*min)							
Baseline	1244 (1083–1403)	233	1264 (1116–1408)	111	1231 (1061–1403)	122	0.57
Cycle 3^b^	-		-		1424 (1151–1662)**	98	-
Follow up	1415(1189–1703)**	114	1345 (1183–1640)*	61	1482 (1175–1792)**	53	0.32
**PPL (sec)**							
Baseline	34.2 (28.5–40.0)	236	34.3 (28.2–39.9)	114	34.2 (28.5–40.2)	122	0.76
Cycle 3	35.3 (28.0–41.3)	195	35.0 (26.2–41.5)	96	35.3 (29.2–39.2)	99	0.92
Follow up	36.9 (30.0–45.9)*	120	36.2 (29.7–45.9)*	63	37.0 (32.0–45.9)	57	0.72
**Total TF (a.u.)**							
Baseline	4.8 (4.6–5.1)	226	4.8 (4.6–5.1)	109	4.8 (4.6–5.2)	117	0.49

IQR = Interquartile range; EV-TF = Tissue factor associated with extracellular vesicles; TG = Thrombin generation; ttPeak = Time to peak; ETP = Endogenous thrombin potential; PPL = Procoagulant phospholipids; TF = Tissue factor; a.u. = Arbitrary units. Changes in biomarker levels from baseline, using Wilcoxon matched-pair signed rank test, noted as:

* unadjusted *P* <0.05

** unadjusted *P* <0.001.

^a^Comparison of biomarker levels based on treatment arms using Mann-Whitney test.

^b^LMWH interferes with TG assay, and the results at cycle 3 are only reported for the control arm.

### Correlations between coagulation biomarkers

**Fig 2:**
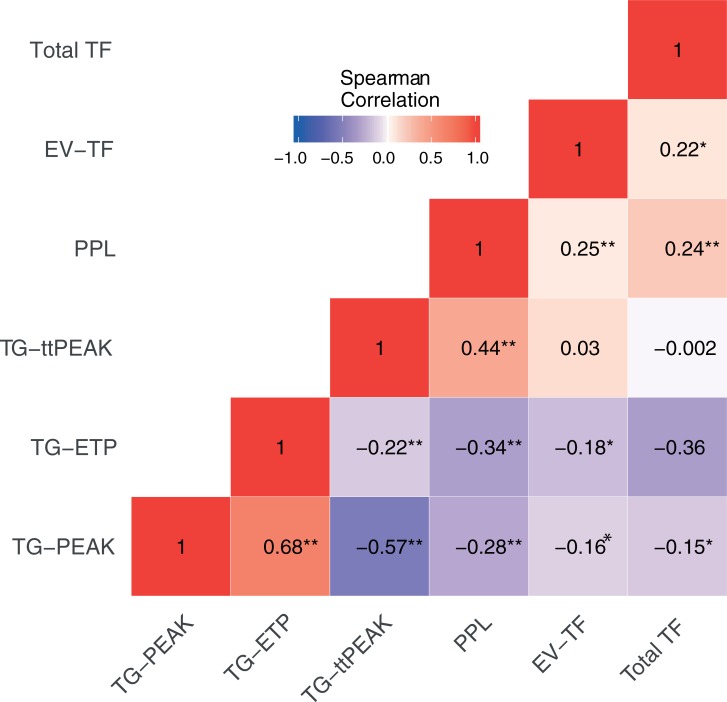
Heat map of biomarker correlations at baseline. TF = Tissue factor; EV-TF = Tissue factor associated with extracellular vesicles; PPL = Procoagulant phospholipids; TG = Thrombin generation; ttPeak = Time to peak; ETP = Endogenous thrombin potential. Please note, TG-ttPeak and PPL are inversely correlated to procoagulant activity, whereas all other biomarkers are positively correlated. * *P* <0.05; ** *P* <0.001.

Expectedly, the strongest correlations were seen between TG-Peak, TG-ETP and TG-ttPeak. There were moderate correlations between the various TG parameters and PPL, while total TF and EV-TF showed weak correlations to other biomarkers (Fig 2).

### Coagulation biomarkers and VTE incidence

In the total patient population, baseline TF was associated with VTE incidence with borderline significance (HR 2.8; *P* = 0.07). In the control arm, patients that eventually were diagnosed with VTE had a small but significantly increased TF level (**Table 3**). EV-TF, TG parameters and PPL did not show any association with VTE risk.

**Table 3:** Coagulation biomarkers at baseline *vs* VTE incidence by treatment arm.

	Control Median (IQR)			LMWH Median (IQR)		
	No VTE *N* = 115	VTE *N* = 12	*P*-Value ^a^	No VTE *N* = 112	VTE^b^ *N* = 3	*P*-Value ^a^
**EV-TF (pg/ml)**	0.18 (0.06–0.31)	0.14 (0.03–0.62)	0.86	0.21 (0.08–0.32)	0.15	0.61
**TG-Peak (nM)**	217 (176–261)	236 (176–277)	0.42	227 (172–267)	199	0.62
**TG-ttPeak (min)**	10.0 (8.4–11.9)	9.1 (7.4–11.2)	0.26	9.8 (8.7–11.2)	9.7	0.87
TG-ETP (nM*min)	1222 (1056–1403)	1336 (1164–1471)	0.26	1271 (1118–1427)	1123	0.15
**PPL (sec)**	34.0 (28.4–40.2)	36.0 (29.4–41.2)	0.88	33.6 (28.0–39.8)	37.1	0.19
**Total TF (a.u.)**	4.8 (4.5–5.1)	5.1 (5.0–5.3)	0.03	4.8 (4.6–5.1)	4.8	0.92

IQR = Interquartile range; VTE = Venous thromboembolism.; EV-TF = Tissue factor associated with extracellular vesicles; TG = Thrombin generation; ttPeak = Time to peak; ETP = Endogenous thrombin potential; PPL = Phospholipids; TF = Tissue factor; a.u. = Arbitrary units

^a^Comparison of biomarker levels based on VTE incidence using Mann-Whitney test.

^b^Due to the limited number of patients in this subgroup data on IQR is not available.

### Coagulation biomarkers and patient outcome

**Fig 3:**
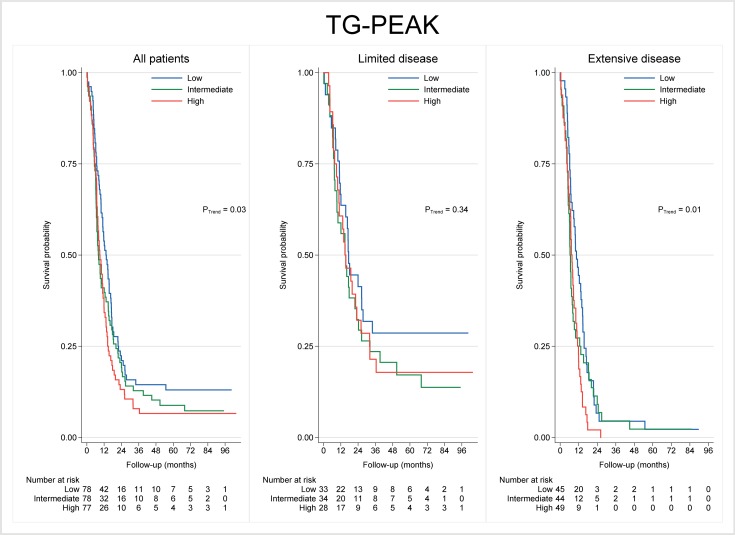
Kaplan-Meier curve of overall survival based on TG-Peak at baseline (tertiles), for all patients and by disease extent.

We next addressed potential associations between baseline values of each coagulation biomarker and patient survival. A significant association was found between increasing TG-Peak levels and decreased OS (*P* = 0.03; **Fig 3** and **Table 4**), and this effect was stronger for patients with ED (*P* = 0.01). For EV-TF, there was a trend towards an association between high levels and decreased OS (*P* = 0.08; **Table** 4).

**Table 4:** Mortality ratios *vs* biomarker levels at baseline, for all patients and by disease stage.

	All patients			Limited disease			Extensive disease		
	HR	95% CI	*P*-value	HR	95% CI	*P*-value	HR	95% CI	*P*-value
**EV-TF**									
Low	1.00			1.00			1.00		
High	1.30	0.97–1.74	0.08	1.20	0.67–2.15	0.53	1.02	0.72–1.44	0.93
**TG-Peak**									
Low	1.00			1.00			1.00		
Intermediate	1.29	0.92–1.80	0.14	1.41	0.82–2.44	0.22	1.30	0.85–1.99	0.22
High	1.44	1.03–2.01	0.03	1.31	0.73–2.33	0.36	1.69	1.11–2.57	0.01

HR = Hazard Ratio; CI = Confidence interval; EV-TF = Tissue factor associated with extracellular vesicles; TG = Thrombin generation; Cut-offs: Upper quartile for EV-TF and tertiles for TG-Peak.

### Coagulation biomarkers and effect of LMWH treatment

**Fig 4:**
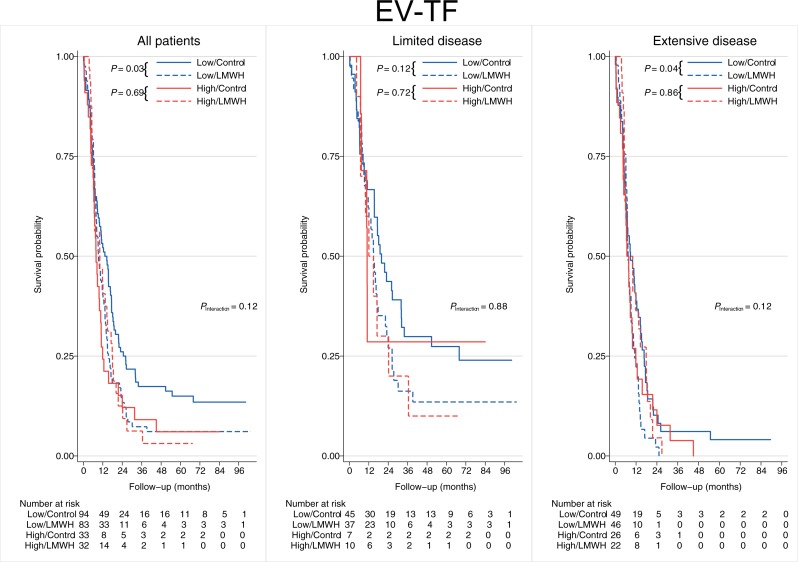
Kaplan-Meier curve of the predictive values of baseline EV-TF (cut-off at the 75th percentile) on the effect of LMWH on overall survival, for all patients and by disease extent.

We next investigated the potential of coagulation parameters to predict the treatment effect of LMWH. Notably, low baseline EV-TF predicted a decreased OS survival in patients receiving the addition of LMWH as compared with control patients receiving chemotherapy only (HR 1.42; 95% confidence interval (CI) 1.04–1.95; *P* = 0.03). The *P* for interaction between baseline EV-TF and survival effect of LMWH was 0.12 and 0.06 for OS with full and 1-year follow-up, respectively, and the effect was specifically seen in patients with ED (Fig 4 and S1 Fig).

## Discussion

The RASTEN study is the largest trial on the survival effect of LMWH in a patient population with a homogenous tumor histology, SCLC [27]. The present *ad hoc* RASTEN sub-study investigated a potential role of biomarkers that reflect various facets of systemic coagulation activity in the prediction of VTE risk and survival in a well-defined patient population enrolled in a clinical trial. Several retrospective studies have investigated potential associations between levels of EV-TF and VTE in *e*.*g*. pancreatic, gastric, lung, brain and ovarian cancer [15–18]. Whereas no such correlations have been found in other cancers, the results with pancreatic cancer are conflicting; Thaler *et al*. [16] did not find any such association in a cohort of 43 patients, whereas Khorana *et al*. reported an association in a cohort encompassing 10 patients [15]. The findings of the present study, showing no association between EV-TF and VTE in SCLC lend further support to the limited value of EV-TF to predict VTE risk in cancer patients.

The Vienna Cancer and Thrombosis Study (CATS) and the HYPERCAN study proposed TG as a marker of the hypercoagulable profile in patients with cancer [8, 9]. However, both studies encompass patients with mixed cancer diagnoses. In our study, with a homogenous trial population, none of the TG parameters predicted VTE risk. These inconsistencies may relate to insufficient standardization of the assays used, but probably also reflect the complexity of the coagulation system in malignancy, *e*.*g*. it remains unclear how key regulatory factors, including antithrombin and tissue factor pathway inhibitor-1, either soluble or EV-associated, contribute to the overall coagulation status in cancer patients.

We found a weak correlation between levels of total TF and EV-TF, which possibly relates to differences between immunologic methods and coagulation activity assays. Further, previous studies showed that immunologic methods have different specificities and may include non-specific binding [31,32]. TF can be in an active or in an inactive state depending on its membrane phospholipid environment and status of a specific disulfide bond in the extracellular domain [33,34]. Immunoassays detect both active and inactive TF whereas EV-TF assays detect only active TF.

Of potential interest, we found evidence of an association of TG-Peak with survival, and EV-TF showed a weak association although neither of the biomarkers associated with VTE risk. It may be speculated that this relates to the procoagulant signaling function of TF and thrombin that can promote tumorigenesis via activation of protease-activated-receptors (PARs) independently of clinically manifest VTE [35]. In this scenario, preferentially EV-associated TF would execute the signaling function through PARs, resulting in *e*.*g*. increased tumor angiogenesis [36]. Another finding of potential interest, was that low baseline EV-TF predicted a negative effect of LMWH treatment on patient survival. With its pleiotropic interactions, including coagulation factors, growth factors, and cytokines, the net effect of LMWH is probably the sum of pro- and anti-tumorigenic activities, where the latter may include inhibition of EV-TF dependent PAR activation [27,37].

Some limitations of our study should be noted. Firstly, few thrombotic events were registered, and VTE data need to be confirmed by further studies in independent cohorts. However, it may be concluded that there is no strong correlation between studied biomarkers and VTE. Secondly, although patient samples were collected according to a well-defined study protocol and each coagulation assay was performed by the same qualified personnel, samples were retrieved from many different sites with potential variations.

To conclude, we found no value of analyzed plasma coagulation biomarkers in the prediction of VTE risk in SCLC, whereas data suggested a potential association between coagulation activation and SCLC aggressiveness. Future studies are warranted, particularly focusing on aspects of the coagulation system in the local tumor microenvironment of SCLC and how this may correlate with VTE and patient outcome.

## Supporting information

S1 Table Biomarkers at baseline by disease extent. (DOCX)

S1 Fig Kaplan-Meier curve of the predictive values of baseline EV-TF (cut-off at the 75th percentile) on the effect of LMWH on 1-year survival, for all patients and by disease extent. (EPS)
