# Supplementary material for: Coagulation biomarkers and prediction of venous thromboembolism and survival in small cell lung cancer: A sub-study of RASTEN - A randomized trial with low molecular weight heparin
Source: PLoS One. 2018 Nov 9;13(11):e0207387. doi: 10.1371/journal.pone.0207387 (PMC6226210; doi:10.1371/journal.pone.0207387)
Supplement: S1 Table — (DOCX) [file pone.0207387.s001.docx]

**Supplementary Table 1.** Biomarkers at baseline by disease extent

|  | Limited disease | | Extensive disease | |  |
| --- | --- | --- | --- | --- | --- |
|  | Median (IQR) | *N* | Median (IQR) | *N* | *P*-value^#^ (LD *vs* ED) |
| **EV-TF (pg/ml)** | 0.16 (0.07-0.27) | 97 | 0.21 (0.08-0.38) | 138 | 0.04 |
| **TG-Peak (nM)** | 218 (177-251) | 95 | 226 (173-276) | 138 | 0.35 |
| **TG-ttPeak (min)** | 9.7 (8.5-11.0) | 95 | 10.0 (8.6-12.0) | 138 | 0.33 |
| **TG-ETP (nM*min)** | 1264 (1118-1403) | 95 | 1239 (1074-1405) | 138 | 0.61 |
| **PPL (sec)** | 35.6 (28.6-41.1) | 96 | 33.3 (28.0-39.6) | 140 | 0.29 |
| **Total TF (a.u.)** | 4.9 (4.6-5.1) | 91 | 4.8 (4.5-5.1) | 135 | 0.34 |

IQR=interquartile range; LD=Limited disease; ED=Extensive disease; EV-TF=Tissue factor associated with extracellular vesicles; TG=Thrombin generation; ttPeak=Time to peak; ETP=Endogenous thrombin potential; PPL=Phospholipids; TF=Tissue factor; a.u. = Arbitrary units

^#^Comparison of biomarker levels based on disease extent using Mann-Whitney test.
